# Derivative of Extremophilic 50S Ribosomal Protein L35Ae as an Alternative Protein Scaffold

**DOI:** 10.1371/journal.pone.0170349

**Published:** 2017-01-19

**Authors:** Anna V. Lomonosova, Andrei B. Ulitin, Alexei S. Kazakov, Tajib A. Mirzabekov, Eugene A. Permyakov, Sergei E. Permyakov

**Affiliations:** 1 Institute for Biological Instrumentation of the Russian Academy of Sciences, Pushchino, Moscow region, Russia; 2 Antherix, Pushchino, Moscow region, Russia; 3 Biomirex Inc., Watertown, Massachusetts, United States of America; Alexion Pharmaceuticals Inc, UNITED STATES

## Abstract

Small antibody mimetics, or alternative binding proteins (ABPs), extend and complement antibody functionality with numerous applications in research, diagnostics and therapeutics. Given the superiority of ABPs, the last two decades have witnessed development of dozens of alternative protein scaffolds (APSs) for the design of ABPs. Proteins from extremophiles with their high structural stability are especially favorable for APS design. Here, a 10X mutant of the 50S ribosomal protein L35Ae from hyperthermophilic archaea *Pyrococcus horikoshii* has been probed as an APS. A phage display library of L35Ae 10X was generated by randomization of its three CDR-like loop regions (repertoire size of 2×10^8^). Two L35Ae 10X variants specific to a model target, the hen egg-white lysozyme (HEL), were isolated from the resulting library using phage display. The affinity of these variants (L4 and L7) to HEL ranges from 0.10 μM to 1.6 μM, according to surface plasmon resonance data. While L4 has 1–2 orders of magnitude lower affinity to HEL homologue, bovine α-lactalbumin (BLA), L7 is equally specific to HEL and BLA. The reference L35Ae 10X is non-specific to both HEL and BLA. L4 and L7 are more resistant to denaturation by guanidine hydrochloride compared to the reference L35Ae 10X (mid-transition concentration is higher by 0.1–0.5 M). Chemical crosslinking experiments reveal an increased propensity of L4 and L7 to multimerization. Overall, the CDR-like loop regions of L35Ae 10X represent a proper interface for generation of functional ABPs. Hence, L35Ae is shown to extend the growing family of protein scaffolds dedicated to the design of novel binding proteins.

## Introduction

Development of proteins capable of specific recognition of biological targets has numerous applications in biotechnology, diagnostics, therapy and research [1–13]. Though antibodies are traditionally used for these purposes [10–12], they suffer from several fundamental disadvantages related to their complex architecture (multi-subunit structure and abundance of post-translational modifications), including limited tissue penetration and access to antigen grooves, need for use of expensive eukaryotic expression systems, and the complicated process of their structural characterization. Antibody alternatives, such as small antibody mimetics, alternative binding proteins (ABPs), based on immunoglobulin-like or non-immunoglobulin folds (‘alternative protein scaffolds’, APSs) have the potential to address these shortcomings [1–9, 13]. An APS possesses a compact stable backbone supporting the target-binding regions, which are genetically randomized to provide a wide repertoire (10^5^−10^13^) of variants with retained structural stability. The resulting combinatorial library serves as a source of proteins specific to a target of choice for *in vitro* display technologies, which give rise to ABPs possessing antibody-like specificity and selectivity to the target [1–9, 13]. The lower structural complexity of ABPs (single subunit structure and minimal post-translational modifications) enables the use of bacterial expression systems, providing higher protein yields and lower production costs, and facilitates their structural characterization. Furthermore, the smaller sizes of ABPs provide efficient tissue penetration, facilitate access to antigen grooves and clefts [13, 14], and promote more selective site blocking in extended targets. The greatly limited serum half-life of ABPs is favorable for tumor imaging and can be extended for therapeutic use by fusion of ABPs with high molecular weight compounds or other half-life increasing entities [7, 8]. ABPs fused with Fc domain attain natural effector functions of antibodies [13]. Finally, ABPs are advantageous for design of multivalent or multispecific molecules [7, 8]. The properties of ABPs, which bridge those of antibodies and low molecular weight drugs/substances, and the ease of modifying ABPs to various applications, guarantee their growing use in resolution of critical problems in biotechnology, medicine and research.

More than 50 [15] APSs have been proposed to date [1–9, 13], numerous ABPs are in clinical trials for treatment of neoplastic, autoimmune, inflammatory, infectious, and ophthalmological diseases [8, 9, 16–19], and one ABP, ecallantide (KALBITOR^®^), has already reached pharmaceutical market. Although several APSs (such as 10^th^ human fibronectin type III domain, Fc-binding Z domain derived from staphylococcal protein A, lipocalins and ankyrin fold) are already broadly established APSs, the natural process of evolution of artificial binding proteins will witness extension of their applications, polishing of validated APSs and development of novel protein scaffolds with superior properties.

**Fig 1:**
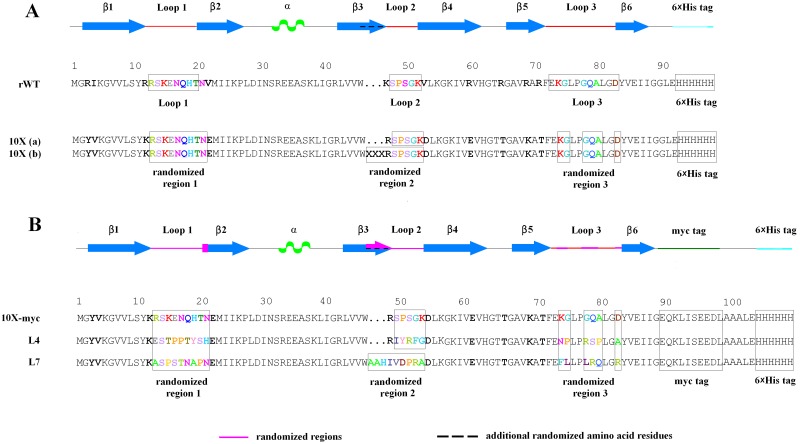
Amino acid sequences for the recombinant forms of L35Ae from *P*. *horikoshii* used in this study. Secondary structure elements of L35Ae from *P*. *furiosus* are indicated (refer to PDB entry 2lp6 [34]): β-sheets 1–6, CDR-like loops 1–3, α-helix (green). The residues affected by randomization are shown in color. **(A)** The amino acid sequences of rWT L35Ae and its 10X mutant [30]. The residues differing between 10X and rWT L35Ae are indicated using bold font. Two 10X variants were used in the phage display library, which contain a loop 2 of original length **(a)** or elongated by three residues **(b**). **(B)** The amino acid sequences of L35Ae 10X with C-terminal GLE sequence replaced by myc tag (‘10X-myc’) and those for HEL-specific binders L4 and L7, isolated from the phage display library of L35Ae 10X. The regions subjected to randomization are indicated in pink.

**Fig 2:**
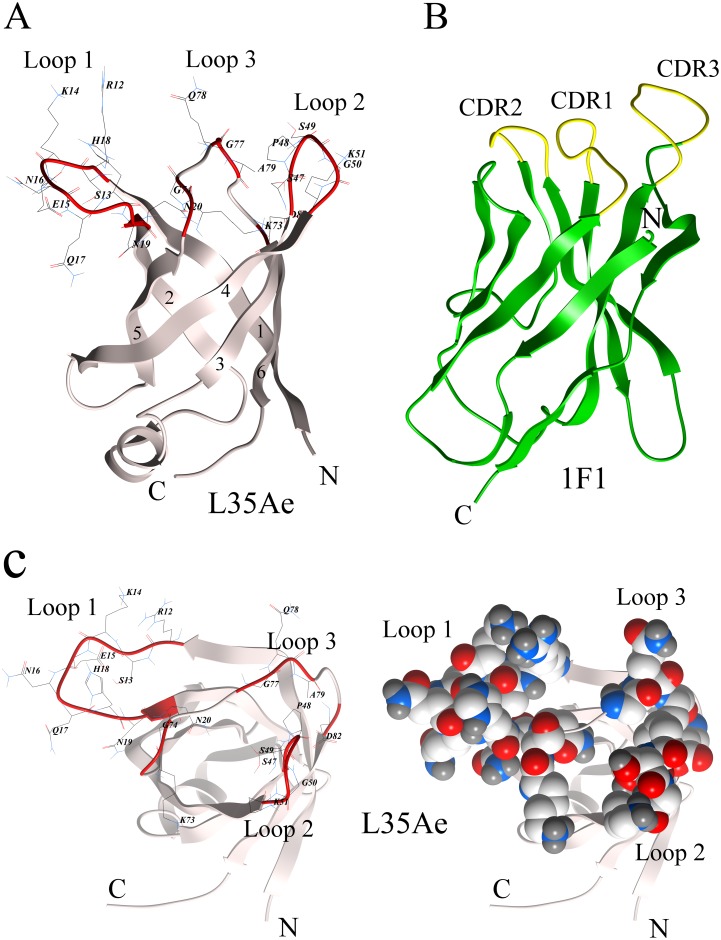
Tertiary structures of 50S ribosomal protein L35Ae from *Pyrococcus furiosus* (sequence identity to L35Ae from *P*. *horikoshii* is 93%; PDB entry 2lp6 [34]) and heavy chain variable region of 1F1 antibody to the influenza A virus hemagglutinin (PDB entry 4gxv [39]). The figure was created using ICM Browser v.3.7-3b (MolSoft L.L.C.) software. **(A)** The β-sheets from 1 to 6 and CDR-like loops 1 to 3 are indicated. The residues randomized in the phage display library of L35Ae 10X (Fig 1) are shown using wire representation (backbone is indicated in red). **(C)** Wire (left) and space-filling (right) representations of top view of the L35Ae structure shown in panel A.

One of the key characteristics of a protein scaffold is its ability to resist multiple amino acid substitutions, deletions and insertions in their ‘paratopic’ regions [20, 21]. Proteins originating from extremophilic organisms are especially attractive in this sense due to their remarkably high structural stability [22–24]. Indeed, Sso7d and Sac7d proteins from thermoacidophilic archaea *Sulfolobus solfataricus* and *Sulfolobus acidocaldarius*, respectively, have been successfully used for engineering of specific binders [25–28]. We have recently shown that 10X mutant (Fig 1A) of 50S ribosomal protein L35Ae from hyperthermophilic archaea *Pyrococcus horikoshii* [29] has several features favoring its use as a protein scaffold [30]: small size (88 residues), lack of disulfides, high thermal stability (mid-transition temperature of 90°C), efficient bacterial production (60 mg of protein per liter of cell culture), lack of non-specific binding to model human embryonic kidney 293 cells (HEK293), and the presence of three nearby loops closely resembling complementarity determining regions (CDRs) of immunoglobulins as judged from tertiary structure of L35Ae from *P*. *furiosus* (Fig 2A and 2C). The intact L35A is located within large ribosomal subunit, associates with initiator and elongator tRNAs [31], affects maturation of 28S and 5.8S rRNAs, biogenesis of large subunit, cell resistance to stress factors, cell proliferation and survival [32, 33]. The tertiary structure of L35Ae from *P*. *furiosus* reveals a six-stranded antiparallel β-barrel with a short α-helix located between strands β2 and β3 with CDR-like loops between strands β1 and β2, β3 and β4, β5 and β6 (referred to as loops 1, 2 and 3, respectively—Figs 1 and 2) forming an extended nearly flat surface potentially suited for target recognition. The proposed on the basis of molecular modelling tRNA-binding site of L35Ae (involves strands β1, β2 and β5 [34]–Fig 2A) was impaired in L35Ae 10X mutant by suppression of its excessive positive charge (Fig 1A), which eliminated L35Ae interaction with surface of HEK293 cells [30].

As opposed to the five-stranded β-barrel of OB-fold proteins [25–28] and eight-stranded β-barrel of Anticalins^™^ [35, 36], to our knowledge, the six-stranded β-barrel found in L35Ae has not been verified as an APS. The CDR-like loops 1 to 3 of L35Ae could serve as a first iteration for search of the protein regions most suited for randomization, since loop diversification of analogous regions has been successfully used for design of ABPs based on β-barrel (OB-fold proteins [25–28], Anticalins^™^ [35, 36]) and β-sandwich folds (Adnectins^™^ [37, 38], *etc*. [5]). Here we employ a phage display technology to probe L35Ae 10X as a protein scaffold for recognition of a model target, hen egg-white lysozyme (HEL), by means of diversification of the CDR-like loop regions of L35Ae 10X.

## Materials and Methods

### Materials

SS320 (MC1061F') electrocompetent cells (a non-amber suppressor strain [F\'*proAB*^+^*lacI*^*q*^*lacZ*ΔM15 *Tn10* (tet^r^)] *hsdR mcrB araD139 Δ*(*araABC-leu*)7679 Δ*lac*X74 *galUgalK rpsL thi*) from Lucigen^®^ were used for the phage display library amplification and cloning. TG1 electrocompetent cells (an amber suppressor strain [F\' *traD36 proAB lacI*^*q*^*Z* ΔM15] *supE thi-1* Δ(*lac-proAB*) Δ(*mcrB-hsdSM*)5(*r*_*k*_^-^ *m*_*k*_^-^)) from Lucigen^®^ were used for the phage display library amplification. *E*. *cloni* 10G electrocompetent cells (*E*. *coli* strain optimized by Lucigen^®^ for high efficiency transformation: F^-^ mcr*A* Δ(*mrr-hsd*RMS-*mcr*BC) *end*A1 *rec*A1 ϕ80*d*lacZΔM15 Δ*lac*X74 *ara*D139 Δ(*ara*,*leu*)7697 *gal*U *gal*K *rps*L (Str^R^) *nup*G λ- *ton*A) were used for amplification of plasmids with genes encoding L35Ae 10X variants.

JM109(DE3) strain (*end*A1, *rec*A1, *gyr*A96, *thi*, *hsd*R17 (r_k_^−^, m_k_^+^), *rel*A1, *sup*E44, λ*–*, Δ(*lac-pro*AB), [F´, *tra*D36, *pro*AB, *lac*I^q^ZΔM15], IDE3) from Promega Corporation was used for expression of HEL-specific binders isolated from the phage display library. Hen egg-white lysozyme (HEL), α-lactalbumin from bovine milk (BLA; type III, calcium depleted) and bovine serum albumin (BSA) were from Sigma-Aldrich Co. Protein concentrations were measured spectrophotometrically using molar extinction coefficient at 280 nm calculated according to ref. [40].

KH_2_PO_4_ and Na_2_HPO_4_ and NaOH were from Panreac Química S.L.U. Sodium acetate, Tris, EDTA, o-phenylenediamine, imidazole, PEG 6000 and skimmed milk were from Sigma-Aldrich^®^ Co. Glycine and Tween 20 were purchased from Bio-Rad Laboratories, Inc. Coomassie Brilliant Blue R-250, boric acid and GuHCl were from Merck KGaA. Kanamycin was from Sintez (Kurgan, Russia). NaCl, components of 2YT media, glycerol, NaHCO_3_, SDS and molecular mass markers for SDS-PAGE were from Helicon (Moscow, Russia). NaN_3_ was from Dia-m (Moscow, Russia). MgCl_2_×6H_2_O and PMSF were purchased from Amresco^®^ LLC. IPTG was from Serva Electrophoresis GmbH. DNAse I was from F. Hoffmann-La Roche Ltd. All restriction enzymes, T4 DNA ligase, β-agarase I, recombinant Taq DNA polymerase and dNTPs were from Thermo Fisher Scientific Inc., Herculase II Fusion DNA polymerase was from Agilent Technologies. Horseradish peroxidase conjugated to anti-M13 monoclonal antibody (27942101) and glutaraldehyde were from GE Healthcare. M13KO7 Helper Phage was from New England Biolabs. Profinity IMAC Ni-Charged Resin was provided by Bio-Rad Laboratories, Inc. All buffers and other solutions were prepared using either distilled or ultrapure water. SnakeSkin dialysis tubing (3.5 kDa MWCO) was from Thermo Fisher Scientific Inc. Medium-binding immuno tubes and medium-binding 96 well ELISA microplates for selection rounds were from Greiner Bio-One. Square bioassay dishes were from Corning Inc.

### Methods

#### Analysis of residue conservation for L35Ae proteins

Full-length amino acid sequences of archaeal L35Ae proteins were extracted from UniProt Knowledgebase (UniProtKB) [41] (release 2015_05, April 29, 2015) using the query “(name:l35ae OR gene:rpl35ae) taxonomy:archaea fragment:no”. Canonical sequences of the resulting 43 entries (6 Swiss-Prot entries) were exported into FASTA format and aligned using Clustal Omega v.1.2.1 algorithm, as implemented in EMBL-EBI online service (http://www.ebi.ac.uk/Tools/msa/clustalo/ [42]). Calculations of residue conservation were performed using AMAS algorithm [43], as implemented in Jalview v.2.8.2 software [44].

#### Construction of the phage display library of L35Ae 10X variants

The amino acid residues of L35Ae 10X subjected to random mutagenesis are indicated in Figs 1A and 2 and S1 Fig. Two variants of L35Ae 10X sequence were used (Fig 1A): the one with original length of the loop 2 (‘10X (a)’) and another one with the loop 2 elongated for three amino acid residues (‘10X (b)’). The codon optimized for expression in *E*. *coli* gene of L35Ae 10X [30] with randomized regions coding the CDR-like loops 1–3 was assembled by overlap extension PCRs, using the primers listed in Table 1 (synthesized by Evrogen, Moscow, Russia).

**Table 1:** List of the primers used for construction of the phage display library of L35Ae 10X variants and their cloning (refer to Fig 1 and S1 and S2 Figs for a scheme of the randomized residues). The degenerate nucleotides [45] are indicated in bold font. RVC and MAC triplets of 2L35L1f primer correspond to residues S14 and N21 of L35Ae 10X.

Primer designation	Nucleotide sequence*
**N36L35NcoPlb-frag**	GTTTTGTTTTCCATGGCGGGTTACGTTAAAGGC
**1L35df**	GTTTGTTTGTTTGAGCTCCGGTTACGTTAAAGGCGTGGTGCTGAGCTATAAG
**2L35L1f**	GTGGTGCTGAGCTATAAG**NNKRV**C**NNKNNKNNKNNKNNKNNKM**ACGAGATGATTATCAAGCCG
**4L35nb**	CCAGACCACCAGGCGGCCAATCAGTTTGCTCGCTTCCTCTCTAGAGTTAATGTCCAGCGGCTTGATAATCATCTCGT
**5L35Lnf**	GGCCGCCTGGTGGTCTGG**NNKNNKNNKNNKNNKNNKNNKNNKNNK**GACCTGAAAGGCAAAATCGTG
**6L35Lnf**	GCCTGGTGGTCTGGAGA**NNKNNKNNKNNKNNK**GACCTGAAAGGCAAAATCGTG
**8L35Lnb**	GAAGGTCGCTTTCACCGCGCCAGTGGTGCCATGCACTTCCACGATTTTGCCTTTCAGGTC
**9L35nb**	GCCGATGATTTCCACGTA**MNN**GCCCAG**MNNMNNMNN**CGGCAG**MNNMNN**TTCGAAGGTCGCTTTCACCGC
**12ExL35nb**	GTGTGTTTGTTGCGGCCGCACCGATGATTTCCACGTA
**L35nMyc-tag**	AGTTTCTGTTCACCGATGATTTCCACGTA
**L35nMyc-tagNotEx**	GTTTGTTAGTTGCGGCCGCCAGATCTTCTTCGCTAATCAGTTTCTGTTCACCGATG
**N36L35NcoMet**	GTTTTTCCATGGGTTACGTTAAAGGC

*‘N’ represents any base, ‘V’ stands for A or C or G, ‘M’ means A or C, ‘K’ corresponds to G or T, and ‘R’ designates A or G [45]

**Fig 3:**
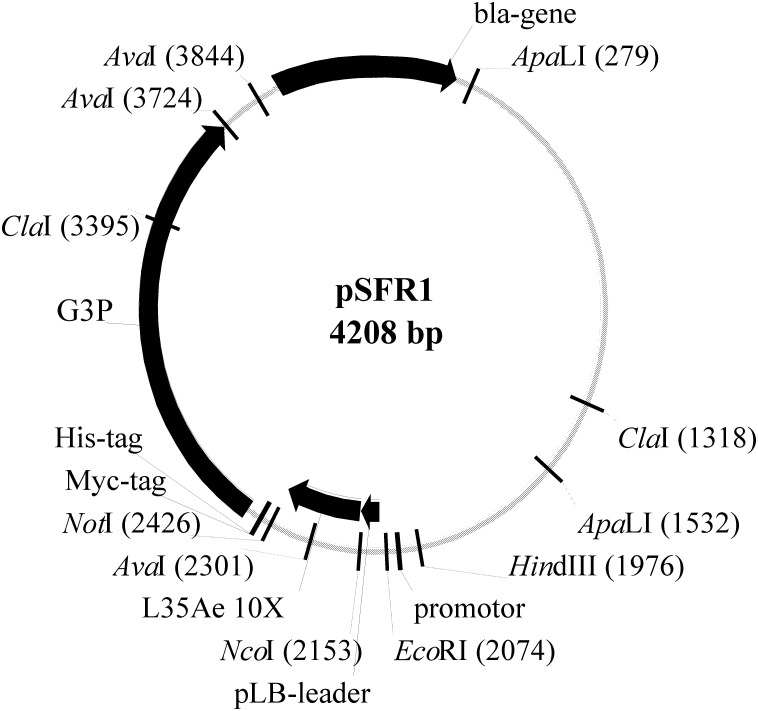
A schematic map of the pSFR1 phagemid (Antherix, Pushchino, Russia) used for phage display of L35Ae 10X. The phagemid is based on pSMART LC Amp vector (Lucigen^®^). Ampicillin resistance of a bacterial host is ensured by the β-lactamase gene, ‘*bla-gene’*. The gene of the 10X mutant of L35Ae from *P*. *horikoshii* was codon optimized for expression in *E*. *coli* [30], subjected to randomization of the regions coding the CDR-like loops 1–3 (Fig 1A, S1 Fig) and cloned between the *Nco*I and *Not*I restriction sites. The L35Ae gene is followed by a myc tag (‘*Myc-tag*’), a 6×His tag (‘*His-tag*’) and the gene of the attachment protein G3P from Enterobacteria phage M13 (‘*G3P*’). The translated chimera of L35Ae 10X-myc (Fig 1B) and G3P is secreted due to the presence of a N-terminal pelB leader sequence (‘*pLB-leader*’).

25 μl of PCR mixture used contained 5 pmol of a pair of primers (2L35L1f - 4L35nb, 5L35Lnf - 8L35Lnb (10X (b) variant) or 6L35Lnf - 8L35Lnb (10X (a) variant)), 2.5 μl of 10× Taq DNA polymerase PCR buffer, 1.25 units of Taq DNA polymerase, 0.25 mM dNTPs. The conditions were: 94°C for 3 min; 10 cycles of 20 s at 94°C, 15 s at 55°C, and 30 s at 72°C; 5 min at 72°C. The resulting PCR products were used as templates for the subsequent PCRs. 50 μl of the reaction mixtures contained 10 pmol of a pair of the primers (1L35df - 4L35nb, 5L35Lnf - 9L35nb (10X (b) variant) or 6L35Lnf - 9L35nb (10X (a) variant)), 5 μl of the template, 4.6 μl of 10× Taq DNA polymerase PCR buffer, 2.5 units of Taq DNA polymerase, 0.25 mM dNTPs. The PCR conditions: 94°C for 3 min; 12 cycles of 25 s at 94°C, 10 s at 55°C, and 30 s at 72°C; 5 min at 72°C. The resulting PCR products were used as templates for the final PCRs. 250 μl of the reaction mixtures contained 50 pmol of the primers (N36L35NcoPlb-frag and 12ExL35nb), 20 μl of a pair of the templates, 50 μl of 5× Herculase II reaction buffer, 12.5 units of Herculase II Fusion DNA polymerase, 0.25 mM dNTPs. The PCR conditions: 94°C for 3 min; 15 cycles of 25 s at 94°C, 25 s at 60°C, and 30 s at 72°C; 5 min at 72°C. The resulting PCR products corresponding to 10X (a) and 10X (b) variants were gel-purified, treated with β-agarase I and digested with *Nco*I and *Xho*I enzymes, followed by their cloning in equimolar ratio into the phagemid based on pSMART LC Amp vector (Lucigen^®^) (Fig 3) predigested by *Nco*I and *Not*I. The ligated resulting pSFR1 phagemid was transformed (Gene Pulser Xcell^™^ Total System, Bio-Rad Laboratories, Inc.) into SS320 (MC1061F') electrocompetent cells according to the supplier’s instructions. The transformed cells were transferred to 50 ml of 2YT medium supplemented with 1% glucose and 10 mM MgCl_2_. After incubation at 37°C for 30 min under gentle shaking, the cells were diluted by 0.6 l of 2YT medium with 100 μg/ml ampicillin and 4×10^9^ particles/ml of M13KO7 Helper Phage and incubated at 37°C for 1 h without stirring. Then 30 μg/ml kanamycin and 0.1 mM IPTG were added, and the phage display library was amplified overnight at 26–27°C under shaking at 250 rpm, isolated using PEG/NaCl precipitation method [46], and stored at -20°C in 20 mM Tris-HCl, 10 mM EDTA, 50% glycerol, 0.1% NaN_3_, pH 7.5 buffer.

#### Isolation of HEL-specific binders from the phage display library of L35Ae 10X

***Pre-depletion of the phage display library***: The L35Ae 10X library was depleted against skimmed milk, used for blocking during the subsequent selection rounds. 2% skimmed milk in PBS, pH 7.0 (MPBS), was adsorbed on the surface of a medium-binding immuno tube by overnight incubation with 4 ml of MPBS at 4°C. The immuno tube was triply washed with PBS, filled with 4 ml of the library solution (2×10^12^ phage particles) in PBST, and rotated at 60 rpm for 1 h at room temperature.

***Phage display selection of HEL-specific L35Ae 10X variants***: The pre-depleted phage display library of L35Ae 10X (see above) was screened for HEL-specific binders by three solid phase selection rounds. The first and second selection rounds were carried out in medium-binding immuno tubes coated with HEL (overnight incubation at 4°C with 4 and 1 ml of 10 and 5 μg/ml HEL in 0.1 M NaHCO_3_ pH 9.6 buffer, respectively). The third selection round was performed in a well of medium-binding ELISA microplate coated with HEL (overnight incubation at 4°C with 0.2 ml of 1 μg/ml HEL in 0.1 M NaHCO3 pH 9.6 buffer). The plastic surface with immobilized HEL was washed with PBS and blocked for 1 h at room temperature with 2% MPBS (rounds 1 and 3) or 1% bovine serum albumin in PBS, pH 7.0 (BPBS, round 2). The blocked surface was triply washed with PBST and incubated at room temperature under the following conditions: incubation in selection round 1 for 2 h (under rotation) with 4 ml of the library solution (5×10^11^ particles/ml) in 2% MPBST; incubation in round 2 for 1 h (under shaking) with 1 ml of the library solution (1.5×10^12^ particles/ml) in 1% BPBST; incubation in round 3 for 1 h (under shaking) with 0.2 ml of the library solution (1×10^12^ particles/ml) in 2% MPBST. Poorly bound phage particles were flushed from the surface with PBST by 10-20-fold rinsing, followed by one PBS wash. Tightly bound phage particles were eluted from the surface by incubation for 5 min with 1 ml (rounds 1–2) or 0.2 ml (round 3) of 0.1 M glycine-HCl, pH 2.5 buffer, followed by immediate solution neutralization by 10–50 μl of 1.5 M Tris-HCl, pH 8.8 buffer. 25 ml of exponentially growing TG1 cell culture (optical density at 600 nm, OD_600_, of 0.5) were infected by the harvested phage particles by incubation at 37°C for 1.5 h without stirring. Infected cell culture was plated on two 2YT 1.2% agar square bioassay dishes supplemented with 100 μg/ml ampicillin and 0.4% glucose. The colonies grown overnight at 30°C were inoculated into 5 ml of 2YT medium and 50 μl of the suspension were inoculated into 25 ml of 2YT medium with 100 μg/ml ampicillin. The cells were grown at 37°C (shaking at 250 rpm) until OD_600_ reached 0.4, then incubated with 4×10^9^ particles/ml of M13KO7 Helper Phage at 37°C for 1.5 h without stirring. After addition of 25 ml of 2YT medium with 100 μg/ml ampicillin, 100 μg/ml kanamycin and 0.2 mM IPTG, the infected culture was grown at 30°C for 5 h, shaking at 250 rpm. The phage particles were isolated using PEG/NaCl precipitation method [46] and used for further selection rounds or analysis of their specificity to HEL by ELISA.

***Analysis of specificity of phage particles to HEL***: The enrichment of the phage display library by L35Ae 10X variants specific to HEL during the selection rounds or specificity to HEL of monoclonal anti-HEL phage particles after the round 3 were evaluated by indirect ELISA. A medium-binding ELISA microplate coated with HEL (overnight incubation at 4°C with 50 μl of 1 and 5 μg/ml HEL in 0.1 M NaHCO_3_ pH 9.6 buffer, for ELISA of monoclonal and polyclonal phage particles, respectively) was blocked with 0.5% MPBST for 1 h at room temperature. 50 μl of the library after the selection rounds in 0.5% MPBST (10^12^ phage particles per ml) or 50 μl of bacterial supernatants, containing monoclonal anti-HEL phage particles, were added to the HEL-coated microplate well, serially diluted and incubated for 1 h at room temperature. The nonspecifically bound phage particles were removed by rinsing the wells with PBST (three times) and PBS (once). The bound phage particles were photometrically detected at 493 nm (Multiskan FC, Thermo Fisher Scientific Inc.) using horseradish peroxidase conjugated to anti-M13 monoclonal antibody (diluted 1:2,500 in PBST, 50 μl per well) and o-phenylenediamine as the substrate.

#### Cloning and isolation of L35Ae 10X variants

***Cloning of genes encoding L35Ae 10X variants***: The genes encoding HEL-specific binders selected from the phage display library of L35Ae 10X were cloned from the pSFR1 phagemid (Fig 3) into pET-28b(+) vector (Novagen^®^) with addition of 3’-terminal oligonucleotide coding myc tag (Fig 1B) for protein detection by ELISA. The reference L35Ae 10X with C-terminal myc tag (‘L35Ae 10X-myc’) was also prepared (Fig 1B) by the analogous cloning of L35Ae 10X gene from the original pET-28b(+) vector [30]. 25 μl of the PCR mixture contained 25 ng of the template (pSFR1 phagemid with the anti-HEL L35Ae 10X variant gene or pET-28b(+) vector with L35Ae 10X gene), 5 pmol of the primers (N36L35NcoMet and L35nMyc-tag, Table 1), 2.5 μl of 10× Taq DNA polymerase PCR buffer, 1.25 units of Taq DNA polymerase, 0.25 mM dNTPs. The conditions were: 94°C for 4 min; 25 cycles of 25 s at 94°C, 25 s at 50°C, and 40 s at 72°C; 5 min at 72°C. The resulting PCR product was treated with 10 units of *Dpn*I for 2 h at 37°C and used as a template for the next PCR. 25 μl of the reaction mixture contained 1 μl of the template, 5 pmol of the primers (N36L35NcoMet and L35nMyc-tagNotEx, Table 1), 2.5 μl of 10× Taq DNA polymerase PCR buffer, 1.25 units of Taq DNA polymerase, 0.25 mM dNTPs. The conditions were: 94°C for 4 min; 25 cycles of 25 s at 94°C, 25 s at 55°C, and 40 s at 72°C; 5 min at 72°C. The resulting PCR product was gel-purified, treated with β-agarase I and digested with *Nco*I and *Not*I enzymes, then cloned into pET-28b(+) vector predigested by *Nco*I and *Not*I. The ligated plasmid was then transformed into *E*. *cloni* 10G electrocompetent cells, plated onto 2YT 1.2% agar supplemented with 50 μg/ml kanamycin and 1% glucose for further plasmid extraction.

***Expression and purification of L35Ae 10X variants***: Electrocompetent JM109(DE3) *E*. *coli* cells were transformed with the L35Ae variant (10X-myc, L4 or L7) plasmid and plated on 2YT medium with 1.2% agar, 50 μg/ml kanamycin and 1% glucose. A colony grown at 37°C for 18 h was inoculated into 8 ml of 2YT medium with 50 μg/ml kanamycin and 1% glucose, and grown for 16 h (37°C, shaking at 250 rpm). The resulting culture was inoculated into 800 ml of 2YT medium with 50 μg/ml kanamycin, and grown at 37°C (shaking at 250 rpm) until the OD_600_ value reached 0.5. L35Ae expression was induced by 0.5 mM IPTG. The cells were grown either for 3 h at 37°C (in the case of L35Ae 10X-myc and L4) or overnight at room temperature (L7). The grown cells were harvested by centrifugation at 3,800 × g for 20 min at 4°C. The cell pellets containing L35Ae 10X-myc or L4 were resuspended in 80 ml of lysis buffer (20 mM H_3_BO_3_-NaOH, 300 mM NaCl, 0.5 mM PMSF, 1 mM EDTA, 5 mM β-mercaptoethanol, 0.1% Tween 20, pH 8.8) and disintegrated using a French press (IBI RAS, Pushchino, Russia). The lysate was incubated with 80 units of DNase I (20 mM MgCl_2_) at 37°C for 15 min and centrifuged at 8,200 × g for 25 min at 4°C. Meanwhile, the L7 variant of L35Ae 10X cell pellet was resuspended in 80 ml of 20 mM H_3_BO_3_-NaOH, 300 mM NaCl, 6M GuHCl, pH 8.8 buffer and incubated overnight at room temperature, stirring at 50–100 rpm, followed by solution clearing by centrifugation at 8,200 × g for 25 min. The 6×His-tagged L35Ae 10X variants were extracted from these cleared supernatants using 5 ml of Profinity IMAC Ni-Charged Resin. The supernatant was incubated with the medium for 2 h under gentle shaking at room temperature. The medium was packed into the column, followed by elution of the bound L35Ae protein with 20 mM H_3_BO_3_-NaOH, 300 mM NaCl, 30 mM EDTA, pH 8.8 buffer. In the case of L7, it was subjected to on-column renaturation prior to the elution: the column was sequentially washed with 10-fold volumes of 20 mM H_3_BO_3_-NaOH, 300 mM NaCl, 10 mM imidazole, pH 8.8 buffers with stepwise decreasing GuHCl concentration (6, 5, 4, 3, 2, 1 and 0 M). The L35Ae variants 10X-myc and L4 were exhaustively dialyzed against distilled water (4°C), freeze-dried and stored at -18°C, whilst L7 was dialyzed against 20 mM H_3_BO_3_-NaOH, 300 mM NaCl, pH 8.8 buffer (4°C) and stored at 4°C. Homogeneity of the L35Ae samples was confirmed by 12% Tris-glycine SDS-PAGE and staining with Coomassie Brilliant Blue R-250. The protein concentrations were measured spectrophotometrically using molar extinction coefficients at 280 nm calculated according to ref. [40]: 9,970 M^-1^cm^-1^ for L35Ae 10X-myc and L7, and 12,950 M^-1^cm^-1^ for L4. The yield was 60, 30, and 90 mg of protein per liter of cell culture, for L35Ae 10X-myc, L4, and L7 samples, respectively.

#### GuHCl-induced unfolding of L35Ae 10X variants

Resistance of L35Ae 10X samples to denaturation by GuHCl was studied as described in ref. [30]. The fluorescence of a single tryptophan of L35Ae 10X (3 μM solution in 20 mM H_3_BO_3_-NaOH, 300 mM NaCl, pH 8.8 buffer; 25°C) was excited at 280 nm. The GuHCl-dependent changes in normalized fluorescence intensity at 314 nm (F_314 nm_) or fluorescence emission spectrum maximum position (*λ*_max_) were fitted by Boltzmann function:

$$P=\frac{P_{free}-P_{GuHCl}}{1+e^{([GuHCl]-{\left[GuHCl\right]}_{1/2})/\Delta }}+P_{GuHCl}$$ (1)

Here, *[GuHCl]*_1/2_ is the mid-transition GuHCl concentration, Δ is a factor reflecting width of the transition, while *P*_*free*_ and *P*_*GuHCl*_ are the F_314 nm_/*λ*_*max*_ values corresponding to GuHCl-free and GuHCl-saturated protein forms, respectively.

#### Chemical crosslinking of L35Ae 10X samples

Crosslinking of non-filtered L35Ae 10X samples (0.3–0.9 mg/ml) by 0.05% glutaraldehyde was performed at 37°C for 1 h (20 mM H_3_BO_3_-NaOH, 300 mM NaCl, pH 8.8) as described in ref. [30].

#### Surface plasmon resonance studies

Surface plasmon resonance (SPR) measurements were carried out at 25°C using Bio-Rad ProteOn^™^ XPR36 system and ProteOn GLH sensor chip. The ligand (50–100 μg/ml HEL, BLA or BSA in 10 mM sodium acetate, pH 4.5 buffer) was immobilized on the chip surface (up to 8,000, 15,500 and 14,500 resonance units (RU), respectively) by amine coupling, according to the manufacturer’s instructions. Unreacted activated amine groups on the chip surface were blocked by 1 M ethanolamine solution. Analyte (0.078–10 μM L35Ae 10X-myc or HEL-specific binder L4/L7 in PBST buffer) was flowed over the chip at rate of 30 μl/min for 300 s, followed by flushing with PBST for 1,200 s at the same flowing rate. The double-referenced SPR sensograms were globally fitted according to a *heterogeneous ligand* model, which assumes existence of two populations of the ligand (L_1_ and L_2_) that bind single analyte molecule (A):

$$L_{1}+A\overset{}{\overset{K_{d1}}{\underset{k_{d1}}{↔}}}L_{1}A;L_{2}+A\overset{}{\overset{K_{d2}}{\underset{k_{d2}}{↔}}}L_{2}A$$ (2)

where *K*_*d*_ and *k*_*d*_ refer to equilibrium and kinetic dissociation constants, respectively. The choice of model (2) is justified by purely statistic manner of ligand immobilization on the chip surface, which leads to appearance of the ligand conformations poorly accessible for interaction. *K*_*d*_, *k*_*d*_, *R*_*max*_ (maximum SPR response) and RI (SPR signal change due to difference between refractive indices of the analyte solution and PBST) values were evaluated using Bio-Rad ProteOn Manager^™^ v.3.1 software. The sensor chip surface was regenerated by passage of 0.5% SDS solution for 50 s, followed by PBST flushing.

## Results

### Design of a combinatorial library of L35Ae 10X

The amino acid residues of the CDR-like loops 1–3 of L35Ae 10X protein suited for randomization were chosen considering both geometric factors and evolutionary conservation of these regions. The tertiary structure of its close relative, L35Ae from *P*. *furiosus* (Fig 2A and 2C), was used as a structural model of L35Ae 10X. The analysis of residue conservation for archaeal L35Ae proteins (S1 Fig) reveals different conservation levels of the CDR-like loops 1–3. The low conservation of loops 1 and 2 suggests their resistance to multiple amino acid substitutions. Therefore, both entire loops, and some flanking residues (C-terminal N21 for loop 1 and N-terminal R47 for loop 2; Fig 1A) were subjected to randomization. Moreover, since loop 2 resists elongation for up to three residues (S1 Fig), 10X(b) variant of L35Ae 10X was designed to elongate loop 2 by three residues (Fig 1A). The marked conservation of the CDR-like loop 3 (S1 Fig) indicates its low resistance to mutagenesis. To limit mutagenesis of the most conservative region of the loop 3 (‘LPGQALG’ sequence; S1 Fig), the ‘LP’ and ‘LG’ residues were excluded from randomization (Fig 1A). The resultant ‘paratopic’ region of L35Ae 10X includes two distinct clusters of the residues provided by loop 1 and residues K74-G75 (loop 3), and loop 2 and residues G78-A80/D83 (loop 3), which are spatially separated by residues L76-P77 (Fig 2C). Overall, 20 (10X(a) variant) or 24 (10X(b) variant) residues of loop regions 1–3 were chosen for random mutagenesis (Fig 1A, S1 Fig): 8 residues of loop 1 with flanking C-terminal residue N21, 5 residues of loop 2 and 3 extra N-terminal residues along with R47 (in the case of 10X(b)), and 6 residues of loop 3. The total number of residues chosen for L35Ae randomization is in line with results for loop randomization of β-barrel structure of lipocalins, where the optimal number of randomized residues, which ensures sufficiently complex ‘paratopic’ region without marked redundancy, lies in the range from 16 to 20 [35].

### Construction of the phage display library of L35Ae 10X

The codon optimized for expression in *E*. *coli* gene of L35Ae 10X [30] with randomized regions coding the CDR-like loops 1–3 (Fig 1, S1 and S2 Figs) was assembled from the primers shown in Table 1 using overlap extension PCRs. A degenerate NNK codon was mostly used for the randomization, except for relatively conserved (S1 Fig) residues S14 and N21 of L35Ae 10X (RVC and MAC triplets were used, respectively) [45]. The resulting pool of L35Ae genes was cloned between the *Nco*I and *Not*I restriction sites of the phagemid, based on pSMART LC Amp vector from Lucigen^®^ (Fig 3). The chimera of L35Ae with C-terminal myc and 6×His tags, and attachment protein G3P of Enterobacteria phage M13, G3P (the amber stop codon between the L35Ae and G3P genes was replaced by a triplet, encoding Arg), should be secreted due to the presence of a N-terminal pelB leader sequence. The resulting pSFR1 phagemid was transformed into SS320 (MC1061F') electrocompetent cells, followed by their infection with M13KO7 Helper Phage, amplification and isolation of the phage display library. Quality of the generated library of L35Ae 10X was verified by analysis of 24 individual randomly selected clones. The clones had correct length of the PCR product corresponding to the L35Ae gene (the fragment size lies in-between 300 bp to 400 bp), as judged from agarose gel electrophoresis. Automatic sequencing of the PCR products evidenced that about quarter (5/19) of the sequenced clones had the expected nucleotide sequences (lacked accidental deletions, insertions, substitutions, and stop codons, and possessed diversified regions from random mutagenesis—see S2 Fig). Nucleotide sequences of the ten clones had unexpected errors, which could be due to insufficient quality of the oligonucleotides used for PCR and/or the errors accumulated during PCR. Meanwhile, similar percentages of correct clones were reported for other combinatorial libraries of APSs (for example, 28–65% for NNS/NNN randomization of 10–14 residues [25, 47]). Considering the efficiency of *E*. *coli* transformation with pSFR1 phagemid and that 26% of the L35Ae 10X library clones were correct, the repertoire size of the library is 2×10^8^, a moderate result for a phage display library [48].

### Isolation of HEL-specific binders from the phage display library of L35Ae 10X

**Fig 4:**
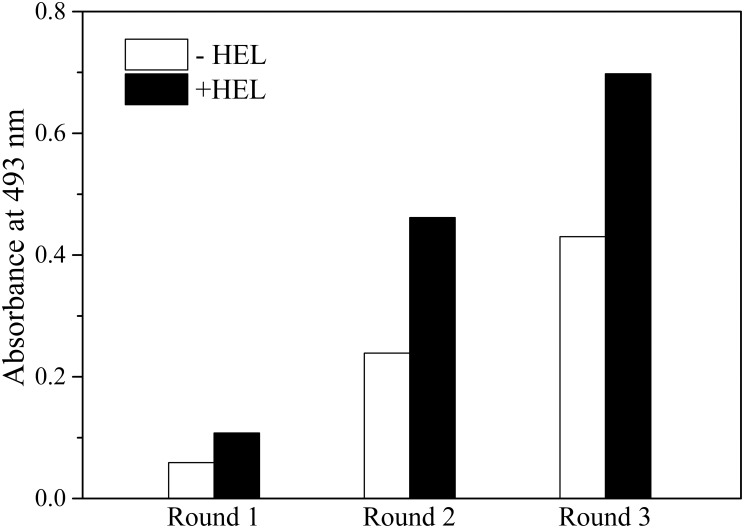
Phage display library enrichment for L35Ae 10X variants specific to HEL during the selection rounds. Specificity to HEL for the polyclonal phage particles after rounds 1–3 was estimated by indirect ELISA. HEL was nonspecifically immobilized on medium-binding ELISA microplate (blocking with 0.5% MPBST); the tightly bound phage particles were detected by absorbance at 493 nm using horseradish peroxidase conjugated to anti-M13 monoclonal antibody and o-phenylenediamine as the substrate. The background ELISA signal was measured using the same protocol, but without HEL immobilization.

Functionality of the L35Ae 10X library was verified by phage display selection of the L35Ae variants specific to a conventional model antigen, HEL. The library was pre-depleted against skimmed milk, used for blocking during the selections. The biopanning process included three selection rounds using HEL nonspecifically adsorbed on medium-binding immuno tubes (rounds 1 and 2) or medium-binding ELISA microplate (round 3). The selection conditions were made increasingly stringent by several methods: decreasing concentrations of both HEL and phage particles, using various blocking agents (2% MPBS or 1% BPBS), shortening the time interval of HEL incubation with the library, and improving washes for poorly bound phage particles on the immobilized HEL. The magnitude of library enrichment by HEL-specific L35Ae 10X variants was evaluated by indirect ELISA during selection rounds. HEL was nonspecifically immobilized on a medium-binding ELISA microplate and the tightly bound phage particles were photometrically detected using horseradish peroxidase conjugated to anti-M13 monoclonal antibody with o-phenylenediamine as the substrate (Fig 4). The specificity of the polyclonal phage particles to HEL gradually increased with iterative selection rounds. Meanwhile, the high background ELISA signal (about half of the signal in the presence of HEL) implies specificity of the phage particles to the blocking agent, MPBST. Since the library was pre-depleted against skimmed milk and incubations of HEL with the library during the selection rounds 1 and 3 were performed in presence of MPBST, the background signals are likely due to homology of some milk components (for example, α-lactalbumin [49]) to HEL.

After the third selection round, 95 individual anti-HEL phage clones were amplified using TG1 cells, and screened for their specificity to HEL using indirect ELISA (data not shown). L35Ae gene sequencing for the eight positive clones have shown presence of two unique anti-HEL L35Ae 10X variants, with loop 2 of the original length, or “L4”, and with loop 2 elongated by three residues, or “L7” (Fig 1B). The residues subjected to randomizations are altered in these variants, except for residues S14 (L4, L7) and N21 (L7) of L35Ae 10X. These residues were randomized in a more limited manner, using RVC and MAC codons, respectively (see Table 1). The HEL-specific clone with an elongated loop 2 developed via biopanning indicates the potential of this loop in generation of novel binding proteins.

### Characterization of the HEL-specific L35Ae 10X variants

The genes encoding L4 and L7 variants of L35Ae 10X were cloned from the pSFR1 phagemid into the pET-28b(+) vector with addition of 3’-terminal oligonucleotide encoding myc tag, facilitating the protein detection by ELISA (Fig 1B). Similarly, the L35Ae 10X gene from the original pET-28b(+) vector [30] was cloned into the pET-28b(+) vector with the addition of a C-terminal extension coding myc tag (encodes the reference ‘L35Ae 10X-myc’ protein—see Fig 1B). The L35Ae 10X variants were expressed in JM109(DE3) *E*. *coli* cells. SDS-PAGE analysis of the protein content in soluble cytoplasmic fraction and inclusion bodies (data not shown) revealed that L35Ae 10X-myc and L4 were expressed mostly in soluble form, while L7 accumulated mainly in inclusion bodies. The inclusion bodies containing L7 were dissolved in 6M GuHCl, followed by the protein incubation with Profinity IMAC Ni-Charged Resin, on-column protein renaturation using stepwise decreasing GuHCl concentration (6, 5, 4, 3, 2, 1 and 0 M), and the protein elution. Other L35Ae variants were also purified to homogeneity in a similar manner, but without the use of GuHCl. Properties of the resulting protein samples crucial to artificial binding protein functionality, including structural stability, susceptibility to multimerization/aggregation, specificity and selectivity of interaction with an antigen, were explored.

**Fig 5:**
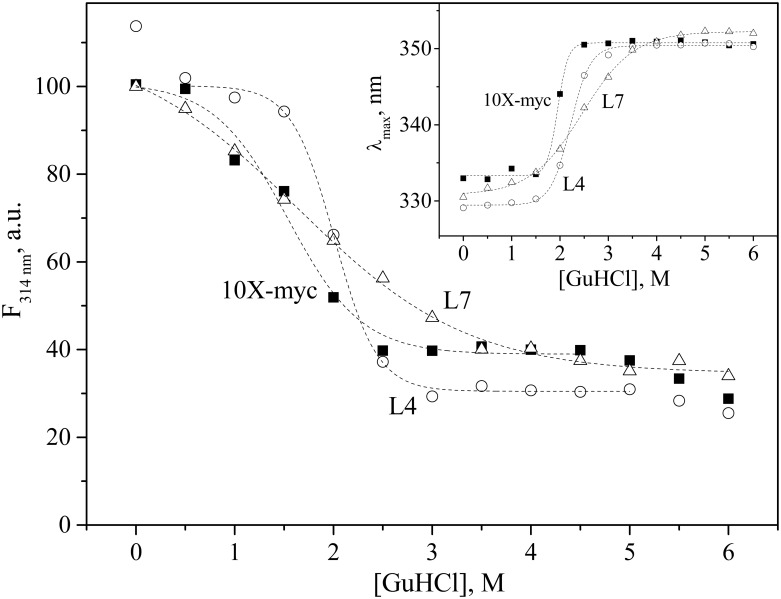
Resistance of anti-HEL binders L4/L7 and L35Ae 10X-myc to GuHCl-induced unfolding monitored by intrinsic fluorescence emission spectroscopy. Excitation wavelength was 280 nm. The normalized fluorescence intensity at 314 nm (F_314 nm_) and fluorescence spectrum maximum position (λ_max_) are shown. Protein concentration was 3 μM. 20 mM H_3_BO_3_, 300 mM NaCl, pH 8.8 buffer; 25°C. The dashed curves are theoretical fits to the experimental data using the Boltzmann function (Eq (1)). The resulting *[GuHCl]*_*1/2*_ values estimated from the F_314 nm_ data: (1.54±0.09) M, (2.01±0.02) M and (1.63±0.10) M for L35Ae 10X-myc, L4 and L7, respectively.

L35Ae 10X variants structural stability was evaluated by resistance to GuHCl-induced unfolding using Trp residue fluorescence emission. The GuHCl-dependent changes in normalized fluorescence intensity at 314 nm and fluorescence spectrum maximum position (Fig 5) demonstrated that the vast mutagenesis of the CDR-like loops 1–3 of L35Ae 10X protein was not accompanied by loss of stability. Instead, L4 and L7 variants exhibited by 0.5 M and 0.1 M higher mid-transition GuHCl concentrations (*[GuHCl]*_*1/2*_), respectively, compared to L35Ae 10X-myc. Overall, the substitution of 22% of L35Ae 10X residues (19/88 of L4 residues) resulted in protein resistance to GuHCl comparable to that of single-chain variable fragments [50]. Hence, structural stability of L35Ae 10X framework is sufficient for resistance to random mutagenesis of loop regions 1–3.

L35Ae 10X variants propensity for multimerization/aggregation was studied by chemical crosslinking of non-filtered samples with glutaraldehyde (Table 2). The predominant form of the studied proteins corresponded to oligomers with molecular mass of 30–100 kDa. Compared to L35Ae 10X-myc, both anti-HEL binders exhibited lower contents of dimers and higher contents of multimers not penetrating the resolving SDS-PAGE gel.

**Table 2:** Distributions of oligomeric forms for various L35Ae 10X variants measured by SDS-PAGE (4–15%) of the proteins, subjected to crosslinking with 0.05% glutaraldehyde at 37°C for 1 h. Buffer conditions: 20 mM H_3_BO_3_, 300 mM NaCl, pH 8.8.

L35Ae 10X variant	Protein concentration, mg/ml	Content of protein forms				
		Monomer, %	Dimer, %	Trimer, %	Oligomers (30–100 kDa), %	High molecular weight fraction *, %
**10X-myc**	0.3	32	23	8	29	8
**L4**		21	13	7	35	25
**L7**		20	13	3	46	18
**10X-myc**	0.9	18	22	8	37	16
**L4**		16	12	7	39	26
**L7**		14	13	4	44	26

* protein oligomers/aggregates not penetrating the resolving 4% gel

**Fig 6:**
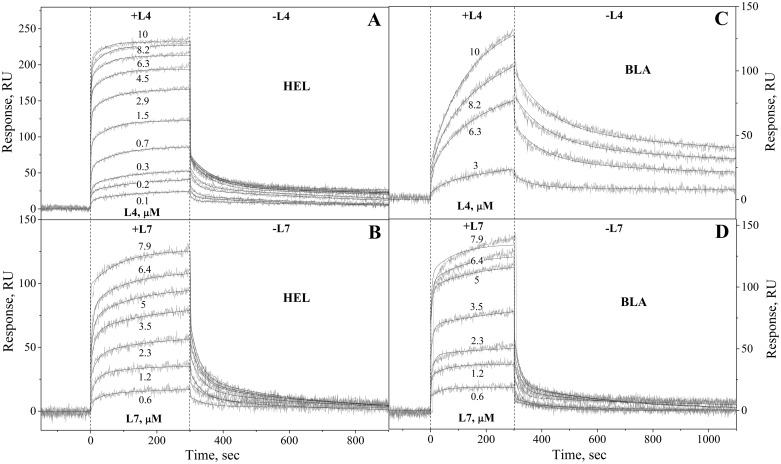
Interaction kinetics for anti-HEL binders L4/L7 with HEL (panels A, B, respectively) or BLA (panels C, D, respectively) at 25°C (PBST buffer), monitored by SPR spectroscopy using HEL/BLA as a ligand. Gray curves are experimental, while black curves are theoretical, calculated according to the heterogeneous ligand model (2) (see Table 3 for the fitting parameters). The analyte concentrations are indicated on the curves in μM.

The specificity and selectivity of interaction of the L35Ae variants with HEL were assessed using SPR spectroscopy. HEL, α-lactalbumin from bovine milk (BLA, a homologue of HEL [49] (sequence identity of 37%), used for assessment of the cross-reactivity) or BSA (negative control) were immobilized on the surface of SPR sensor chip by amine coupling and a set of injections of L4/L7 or L35Ae 10X-myc (negative control) solutions was carried. While no effects were observed for L35Ae 10X-myc at protein concentrations of up to 10 μM (data not shown), and no interaction with BSA was observed for anti-HEL binders L4 and L7 at up to 10 μM concentrations (data not shown), the SPR sensograms for L4/L7 exhibited a characteristic concentration-dependent association-dissociation pattern in the case of HEL and BLA (Fig 6). The kinetic SPR data for the L4-HEL interaction (Fig 6A) are well approximated by the heterogeneous ligand model (2) with equilibrium dissociation constants, *K*_*d*_, of 0.25 μM and 0.10 μM (Table 3). Analogous experiments for the L7-HEL interaction (Fig 6B) gave *K*_*d*_ values of 1.6 μM (Table 3). These estimates were also qualitatively confirmed by ELISA using the Beatty method [51] (data not shown). Meanwhile, both anti-HEL binders exhibited cross-reactivity with BLA: *K*_*d*_ values of 22 μM and 2.1 μM for L4, and 1.9 μM and 2.7 μM for L7 (Fig 6, Table 3). Thus, L7 is equally specific to HEL and BLA, while specificity of L4 to HEL exceeds that to BLA by 1–2 orders of magnitude. Overall, unlike the original L35Ae 10X protein, both L4 and L7 possess submicromolar to micromolar affinity to HEL, and cross-react with its homologue, despite multimerization at high concentrations (Table 2). Cross-reactivity with related proteins was reported for some of novel binding proteins, including those based on DARPin and Sso7d frameworks [26, 52].

**Table 3:** Parameters of the heterogeneous ligand model (2) describing the SPR data on kinetics of interaction between anti-HEL binders L4/L7 and HEL/BLA (see Fig 6).

Analyte	Ligand	Parameter					
		k_d1_, s^-1^	K_d1_	R_max1_	k_d2_, s^-1^	K_d2_	R_max2_
**L4**	HEL	(4.1±1.5)×10^−2^	(2.5±1.1)×10^−7^	36	(9.4±1.5)×10^−4^	(1.0±0.4)×10^−7^	28
	BLA	(1.1±0.4)×10^−2^	(2.2±1.6)×10^−5^	152	(3.3±0.2)×10^−4^	(2.1±0.9)×10^−6^	83
**L7**	HEL	(2.7±0.3)×10^−2^	(1.6±0.9)×10^−6^	35	(1.5±0.3)×10^−3^	(1.6±0.2)×10^−6^	22
	BLA	(4.1±1.0)×10^−2^	(1.9±0.6)×10^−6^	40	(4±2)×10^−3^	(2.7±1.6)×10^−6^	22

## Discussion

The 10X mutant of 50S ribosomal protein L35Ae of the hyperthermophilic archaea, *P*. *horikoshii*, has previously been shown to possess favorable features for protein scaffolding use [30]. Its unique six-stranded β-barrel motif is not found in existing scaffold proteins, and contains a nearly flat surface with CDR-like loops 1–3, which have target recognition potential (Fig 2). L35Ae 10X’s ability to serve as a framework for alternative binding proteins development was evaluated here via construction of a M13 phage display library of its variants, followed by isolation and characterization of the variants specific to HEL, a conventional model antigen. The residues of L35Ae 10X subjected to randomization form a double ‘paratopic’ region including two clusters of the residues provided by the CDR-like loop 1 and residues K74-G75 of loop 3, and loop 2 and residues G78-A80/D83 of loop 3, which are separated by residues L76-P77 (the model is based on tertiary structure of L35Ae from *P*. *furiosus*–see Fig 2). The analysis of residue conservation for archaeal L35Ae proteins (S1 Fig) shows low conservation of the loops 1 and 2 along with high conservation of the loop 3, which constrains its randomization. On the contrary, loop 2 resists elongation for up to three residues (S1 Fig), which enabled us to design a 10X(b) variant of L35Ae 10X with loop 2 elongated by three residues (Fig 1A). Overall, 20 or 24 residues of the loop regions 1–3 were subjected to randomization (Fig 1, S1 and S2 Figs) using mostly a degenerate NNK codon (see Table 1), covering all 20 amino acids [45]. Apparently, theoretical limit of diversity of such library (up to 10^31^) greatly exceeds the limitations imposed by the use of phage display technology (library size up to 10^12^ [48]). Diversity of the constructed phage display library of L35Ae 10X reaches 2×10^8^, which corresponds to only 10^−23^ of the theoretical limit. The L35Ae 10X library’s ability to provide binders for a model antigen with *K*_*d*_ values down to 0.1 μM (Table 3) despite sparse sampling of the sequence space is promising. The low coverage of the sequence space could explain low selectivity of L4 and L7 variants to HEL, manifested as their marked affinity to a homologous milk protein, BLA (Fig 6 and Table 3). The observed specificity of L4 and L7 to BLA is unexpected, given that the library was pre-depleted against BLA-containing MPBS and that incubations of HEL with the library during selections were performed in presence of MPBST. This contradiction could be resolved assuming that binding of skimmed milk components to BLA suppresses its affinity to the L35Ae 10X variants. L4 and L7 ‘s cross-reactivity with BLA could explain the high-background ELISA signals observed during examinations of specificity to HEL of the polyclonal phage particles after the selection rounds (Fig 4). Meanwhile, L4 or L7 variants expectedly do not recognize another major component of skimmed milk, BSA. The lack of affinity of reference protein (L35Ae 10X-myc) for HEL, BLA and BSA is an evidence that L35Ae 10X variants specificity and selectivity for HEL/BLA arose due to biopanning.

Comparable to worse results were reported for a pioneer trial of 10^th^ human fibronectin type III domain (^10^Fn3) as a protein scaffold using yeast ubiquitin as a model target [38]. Five selection rounds from M13 phage display library of ^10^Fn3 with NNK randomization of 10 residues of its nearby BC and FG loops (repertoire of 10^8^) gave a single dominant clone with micromolar affinity to ubiquitin (IC_50_ of 5 μM), cross-reactivity with dextran (not used in the selections), lowered structural stability and water solubility. Since then, ongoing development of ^10^Fn3 protein scaffold properties have placed it among the best established scaffolds to date (Adnectins^™^, reviewed in ref. [37]). Given this context, the prospects of L35Ae elaboration for scaffold application seems to be promising.

In general, existing combinatorial libraries of other proteins from extremophiles, such as Sso7d from *S*. *solfataricus* and Sac7d proteins from *S*. *acidocaldarius* [25–28], have been shown to provide more potent target-specific binders (see Table 4 for comparison with the L35Ae 10X library). Compared to the L35Ae-based binders, the binders from Sso7d and Sac7d libraries showed 1–3 orders of magnitude lower limit of *K*_*d*_ values and higher resistance to GuHCl (for the Sso7d-based binders), while being mostly monomeric (Table 4). Meanwhile, the Sso7d and Sac7d libraries have a higher ratio of library size and theoretical limit of the library diversity (R, corresponds to the fraction of the calculated library repertoire, which is used in practice): 10^−5^ and 0.3–3×10^−8^ for the Sso7d and Sac7d libraries, respectively, versus 10^−23^ for the L35Ae 10X library. In this sense, the practical diversity of the L35Ae library is many orders of magnitude more limited, which deteriorates target specificity and selectivity of its binders. Geometric factors of the ‘paratopic’ regions of a scaffold protein are also crucial for efficient recognition of diverse targets. In contrast to the loop randomization used for the L35Ae library, the Sso7d and Sac7d libraries are based on ‘flat surface’ random mutagenesis, and loops are only diversified in some Sac7d libraries. This approach is successfully applied in DARPin, one of the most advanced protein scaffolds to date [53]. Taking these consideration into account, more focused L35Ae 10X loop randomization and combination with ‘flat surface’ randomization, aimed at increased R value and geometrical optimization of the ‘paratopic’ region, and use of more diverse libraries, should favor higher target specificity and selectivity for L35Ae binders. Furthermore, preliminary examination of structural properties of L35Ae proteins from other extremophiles (unpublished data) has demonstrated that some of the proteins exhibit improved properties as compared to L35Ae from *P*. *horikoshii* and its 10X mutant [30] (monomeric structure, considerably higher structural stability, and negligible non-specific binding to model mammalian cells). Hence, use of these proteins as a framework for development of the L35Ae-based protein scaffold could improve both structural stability and susceptibility to multimerization of its binders.

**Table 4:** Comparison of protein combinatorial libraries based on proteins originating from extremophiles, including Sac7d, Sso7d and L35Ae 10X.

Scaffold protein	Number of residues	Library properties			Properties of clones isolated from the library				References
		Number of randomized residues, diversified regions, degenerate codons used	Display method	Library size	Target	K_d_, nM	[GuHCl]_1/2_, M	Propensity to oligomerization	
**L35Ae 10X**	88	20/24, loops, mostly NNK	Phage display	2×10^8^	HEL	100–1600	1.6–2.0	yes	present study
**Sso7d**	63	10, ‘flat surface’, NNN	Yeast display	10^8^	fluorescein, 12 amino acid peptide from the C-terminus of β-catenin, HEL, streptavidin, immunoglobulins G from mouse and chicken [25]; Fc domain of human immunoglobulin [26]	12–7569	2.5–5 [25]	yes/no [25]; no [26]	[25, 26]
**Sac7d**	66	10–15, ‘flat surface’ or ‘flat surface & loops’, mostly NNS	Ribosome display	(1–3)×10^12^	PulD-N fragment [47]; CelD from *Clostridium thermocellum*, HEL [54]; Fc domain of human immunoglobulin G [27]	0.14–98	data not available	no [27, 54]	[27, 47, 54]

## Conclusions

The first trial of extremophilic L35Ae 10X protein and its unique six-stranded β-barrel motif not found in the established scaffold proteins as a source of novel binding proteins is performed here using HEL as a model target. The random mutagenesis of three nearby CDR-like loop regions of the protein, followed by phage display isolation of HEL-specific L35Ae 10X variants, have shown that L35Ae’s structure successfully resists randomization in these protein regions and provides the variants with submicromolar to micromolar affinity to HEL, and marked cross-reactivity with its close homologue, BLA. These results are superior to those reported in a pioneer trial of ^10^Fn3, which is currently among the most advanced scaffold proteins (Adnectins^™^) [37, 38]. Meanwhile, properties of the HEL-specific L35Ae 10X binders are overall inferior to properties of target-specific binders isolated from combinatorial libraries of Sso7d and Sac7d proteins from extremophilic archaea [25–28] (Table 4). Negative features of the HEL-specific L35Ae binders, such as relatively low affinity and selectivity to HEL, relatively low resistance to GuHCl, and increased propensity to multimerization, could be suppressed by further optimization, including use of L35Ae proteins with initially superior properties, combining loop randomization with ‘flat surface’ randomization, use of more focused and more diverse protein libraries. Therapeutic use of L35Ae-based proteins will require addressing the issue of their potential immunogenicity; usage of L35Ae-based binding proteins for research and diagnostics represents a more straightforward path for potential practical application. In summary, though further refinement would be needed for application to practical use, this work shows that L35Ae 10X is a viable scaffold protein ripe for further development.

## Supporting Information

S1 Fig Analysis of residue conservation for archaeal L35Ae proteins (UniProtKB release 2015_05) using AMAS algorithm [43]. The multiple sequence alignment of the L35Ae proteins was performed using Clustal Omega v.1.2.1 algorithm [42]. Loops 1 to 3 of L35Ae from *P*. *furiosus* are indicated (refer to PDB entry 2lp6 [34], Fig 2A and 2C), as well as the residues randomized in the phage display library of L35Ae 10X (designated as ‘+’, see Fig 1).
(TIF)

S2 Fig Assessment of quality for the phage display library of L35Ae 10X variants by automatic sequencing of the PCR product corresponding to the L35Ae gene for the clones with expected nucleotide sequence (lack accidental deletions/insertions/substitutions and stop codons in the sequenced regions and possess diversified regions subjected to random mutagenesis). The multiple sequence alignment of the clones and the analysis of residue conservation were performed using Clustal Omega v.1.2.1 [42] and AMAS algorithms [43], respectively. Loops 1 to 3 of L35Ae from *P*. *furiosus* are indicated (refer to PDB entry 2lp6 [34], Fig 2A and 2C), as well as the residues randomized in the phage display library of L35Ae 10X (designated as ‘+’, see Fig 1).
(TIF)

## References

[1] Hey T, Fiedler E, Rudolph R, Fiedler M. Artificial, non-antibody binding proteins for pharmaceutical and industrial applications. Trends Biotechnol. 2005;23(10):514–22. Epub 2005/08/02. 10.1016/j.tibtech.2005.07.007 16054718

[2] Wurch T, Pierre A, Depil S. Novel protein scaffolds as emerging therapeutic proteins: from discovery to clinical proof-of-concept. Trends Biotechnol. 2012;30(11):575–82. Epub 2012/09/05. 10.1016/j.tibtech.2012.07.006 22944617

[3] Skerra A. Alternative non-antibody scaffolds for molecular recognition. Curr Opin Biotechnol. 2007;18(4):295–304. Epub 2007/07/24. 10.1016/j.copbio.2007.04.010 17643280

[4] Nygren PA, Skerra A. Binding proteins from alternative scaffolds. J Immunol Methods. 2004;290(1–2):3–28. Epub 2004/07/21. 10.1016/j.jim.2004.04.006 15261569

[5] Binz HK, Amstutz P, Pluckthun A. Engineering novel binding proteins from nonimmunoglobulin domains. Nat Biotechnol. 2005;23(10):1257–68. Epub 2005/10/08. 10.1038/nbt1127 16211069

[6] Hosse RJ, Rothe A, Power BE. A new generation of protein display scaffolds for molecular recognition. Protein Sci. 2006;15(1):14–27. Epub 2005/12/24. 10.1110/ps.051817606 16373474 PMC2242358

[7] Jost C, Pluckthun A. Engineered proteins with desired specificity: DARPins, other alternative scaffolds and bispecific IgGs. Curr Opin Struct Biol. 2014;27C:102–12. Epub 2014/07/18. 10.1016/j.sbi.2014.05.011 25033247

[8] Weidle UH, Auer J, Brinkmann U, Georges G, Tiefenthaler G. The emerging role of new protein scaffold-based agents for treatment of cancer. Cancer Genomics Proteomics. 2013;10(4):155–68. Epub 2013/07/31. 23893924

[9] Baloch AR, Baloch AW, Sutton BJ, Zhang X. Antibody mimetics: promising complementary agents to animal-sourced antibodies. Crit Rev Biotechnol. 2014:1–8. Epub 2014/09/30. 10.3109/07388551.2014.958431 25264572

[10] Ribatti D. From the discovery of monoclonal antibodies to their therapeutic application: An historical reappraisal. Immunology Letters. 2014;161(1):96–9. 10.1016/j.imlet.2014.05.010 24877873

[11] Hozumi N, Sandhu JS. Recombinant antibody technology: its advent and advances. Cancer Invest. 1993;11(6):714–23. Epub 1993/01/01. 8221205 10.3109/07357909309046945

[12] Souriau C, Hudson PJ. Recombinant antibodies for cancer diagnosis and therapy. Expert Opin Biol Ther. 2003;3(2):305–18. Epub 2003/03/29. 10.1517/14712598.3.2.305 12662144

[13] Saerens D, Ghassabeh GH, Muyldermans S. Single-domain antibodies as building blocks for novel therapeutics. Curr Opin Pharmacol. 2008;8(5):600–8. 10.1016/j.coph.2008.07.006 18691671

[14] Skerra A. Engineered protein scaffolds for molecular recognition. J Mol Recognit. 2000;13(4):167–87. Epub 2000/08/10. 10.1002/1099-1352(200007/08)13:4<167::AID-JMR502>3.0.CO;2-9 10931555

[15] Gebauer M, Skerra A. Alternative Protein Scaffolds as Novel Biotherapeutics In: Rosenberg A, Demeule B, editors. Biobetters: Protein Engineering to Approach the Curative. New York, NY: Springer New York; 2015 p. 221–68.

[16] Friedman M, Stahl S. Engineered affinity proteins for tumour-targeting applications. Biotechnol Appl Biochem. 2009;53(Pt 1):1–29. Epub 2009/04/04. 10.1042/BA20080287 19341363

[17] Gebauer M, Skerra A. Engineered protein scaffolds as next-generation antibody therapeutics. Curr Opin Chem Biol. 2009;13(3):245–55. 10.1016/j.cbpa.2009.04.627 19501012

[18] Beck A, Wurch T, Bailly C, Corvaia N. Strategies and challenges for the next generation of therapeutic antibodies. Nat Rev Immunol. 2010;10(5):345–52. 10.1038/nri2747 20414207

[19] Wurch T, Pierre A, Depil S. Novel protein scaffolds as emerging therapeutic proteins: from discovery to clinical proof-of-concept. Trends Biotechnol. 2012;30(11):575–82. 10.1016/j.tibtech.2012.07.006 22944617

[20] Skerra A. Lipocalins as a scaffold. Biochim Biophys Acta. 2000;1482(1–2):337–50. Epub 2000/11/04. 11058774 10.1016/s0167-4838(00)00145-x

[21] Bloom JD, Labthavikul ST, Otey CR, Arnold FH. Protein stability promotes evolvability. Proc Natl Acad Sci U S A. 2006;103(15):5869–74. Epub 2006/04/04. 10.1073/pnas.0510098103 16581913 PMC1458665

[22] Vieille C, Zeikus GJ. Hyperthermophilic enzymes: sources, uses, and molecular mechanisms for thermostability. Microbiol Mol Biol Rev. 2001;65(1):1–43. Epub 2001/03/10. 10.1128/MMBR.65.1.1-43.2001 11238984 PMC99017

[23] Ladenstein R, Antranikian G. Proteins from hyperthermophiles: stability and enzymatic catalysis close to the boiling point of water. Adv Biochem Eng Biotechnol. 1998;61:37–85. Epub 1998/07/22. 9670797 10.1007/BFb0102289

[24] Luke KA, Higgins CL, Wittung-Stafshede P. Thermodynamic stability and folding of proteins from hyperthermophilic organisms. FEBS J. 2007;274(16):4023–33. Epub 2007/08/09. 10.1111/j.1742-4658.2007.05955.x 17683332

[25] Gera N, Hussain M, Wright RC, Rao BM. Highly stable binding proteins derived from the hyperthermophilic Sso7d scaffold. J Mol Biol. 2011;409(4):601–16. Epub 2011/04/26. 10.1016/j.jmb.2011.04.020 21515282

[26] Gera N, Hill AB, White DP, Carbonell RG, Rao BM. Design of pH sensitive binding proteins from the hyperthermophilic Sso7d scaffold. PLoS One. 2012;7(11):e48928 Epub 2012/11/13. 10.1371/journal.pone.0048928 23145025 PMC3492137

[27] Behar G, Bellinzoni M, Maillasson M, Paillard-Laurance L, Alzari PM, He X, et al Tolerance of the archaeal Sac7d scaffold protein to alternative library designs: characterization of anti-immunoglobulin G Affitins. Protein Eng Des Sel. 2013;26(4):267–75. Epub 2013/01/15. 10.1093/protein/gzs106 23315487

[28] Mouratou B, Schaeffer F, Guilvout I, Tello-Manigne D, Pugsley AP, Alzari PM, et al Remodeling a DNA-binding protein as a specific in vivo inhibitor of bacterial secretin PulD. P Natl Acad Sci USA. 2007;104(46):17983–8. 10.1073/pnas.0702963104 PMC2084283 17984049

[29] Kawarabayasi Y, Sawada M, Horikawa H, Haikawa Y, Hino Y, Yamamoto S, et al Complete sequence and gene organization of the genome of a hyper-thermophilic archaebacterium, Pyrococcus horikoshii OT3 (supplement). DNA Res. 1998;5(2):147–55. Epub 1998/07/29. 9679203 10.1093/dnares/5.2.147

[30] Lomonosova AV, Ovchinnikova EV, Kazakov AS, Denesyuk AI, Sofin AD, Mikhailov RV, et al Extremophilic 50S Ribosomal RNA-Binding Protein L35Ae as a Basis for Engineering of an Alternative Protein Scaffold. Plos One. 2015;10(8):e0134906 Epub 2015/08/08. 10.1371/journal.pone.0134906 26247602 PMC4527664

[31] Ulbrich N, Wool IG, Ackerman E, Sigler PB. The Identification by Affinity-Chromatography of the Rat-Liver Ribosomal-Proteins That Bind to Elongator and Initiator Transfer Ribonucleic-Acids. Journal of Biological Chemistry. 1980;255(14):7010–6. 7391064

[32] Farrar JE, Nater M, Caywood E, McDevitt MA, Kowalski J, Takemoto CM, et al Abnormalities of the large ribosomal subunit protein, Rpl35a, in Diamond-Blackfan anemia. Blood. 2008;112(5):1582–92. Epub 2008/06/07. 10.1182/blood-2008-02-140012 18535205 PMC2518874

[33] Lopez CD, Martinovsky G, Naumovski L. Inhibition of cell death by ribosomal protein L35a. Cancer Lett. 2002;180(2):195–202. 12175552 10.1016/s0304-3835(02)00024-1

[34] Snyder DA, Aramini JM, Yu B, Huang YJ, Xiao R, Cort JR, et al Solution NMR structure of the ribosomal protein RP-L35Ae from Pyrococcus furiosus. Proteins. 2012;80(7):1901–6. Epub 2012/03/17. 10.1002/prot.24071 22422653 PMC3639469

[35] Richter A, Eggenstein E, Skerra A. Anticalins: exploiting a non-Ig scaffold with hypervariable loops for the engineering of binding proteins. FEBS letters. 2014;588(2):213–8. Epub 2013/11/19. 10.1016/j.febslet.2013.11.006 24239535

[36] Beste G, Schmidt FS, Stibora T, Skerra A. Small antibody-like proteins with prescribed ligand specificities derived from the lipocalin fold. P Natl Acad Sci USA. 1999;96(5):1898–903. Epub 1999/03/03. 10.1073/pnas.96.5.1898 PMC26708 10051566

[37] Lipovsek D. Adnectins: engineered target-binding protein therapeutics. Protein Eng Des Sel. 2011;24(1–2):3–9. Epub 2010/11/12. 10.1093/protein/gzq097 21068165 PMC3003446

[38] Koide A, Bailey CW, Huang X, Koide S. The fibronectin type III domain as a scaffold for novel binding proteins. J Mol Biol. 1998;284(4):1141–51. Epub 1998/12/05. 10.1006/jmbi.1998.2238 9837732

[39] Tsibane T, Ekiert DC, Krause JC, Martinez O, Crowe JE, Wilson IA, et al Influenza Human Monoclonal Antibody 1F1 Interacts with Three Major Antigenic Sites and Residues Mediating Human Receptor Specificity in H1N1 Viruses. Plos Pathog. 2012;8(12). 10.1371/journal.ppat.1003067 PMC3516549 23236279

[40] Pace CN, Vajdos F, Fee L, Grimsley G, Gray T. How to measure and predict the molar absorption coefficient of a protein. Protein Sci. 1995;4(11):2411–23. Epub 1995/11/01. 10.1002/pro.5560041120 8563639 PMC2143013

[41] The Universal Protein Resource (UniProt) in 2010. Nucleic Acids Res. 2010;38(Database issue):D142–8. Epub 2009/10/22. 10.1093/nar/gkp846 19843607 PMC2808944

[42] McWilliam H, Li W, Uludag M, Squizzato S, Park YM, Buso N, et al Analysis Tool Web Services from the EMBL-EBI. Nucleic acids research. 2013;41(Web Server issue):W597–600. Epub 2013/05/15. 10.1093/nar/gkt376 23671338 PMC3692137

[43] Livingstone CD, Barton GJ. Protein-Sequence Alignments—a Strategy for the Hierarchical Analysis of Residue Conservation. Computer Applications in the Biosciences. 1993;9(6):745–56. 8143162 10.1093/bioinformatics/9.6.745

[44] Waterhouse AM, Procter JB, Martin DM, Clamp M, Barton GJ. Jalview Version 2—a multiple sequence alignment editor and analysis workbench. Bioinformatics. 2009;25(9):1189–91. Epub 2009/01/20. 10.1093/bioinformatics/btp033 19151095 PMC2672624

[45] Nomenclature for Incompletely Specified Bases in Nucleic-Acid Sequences—Recommendations 1984. Biochemical Journal. 1985;229(2):281–6. 4038268 10.1042/bj2290281 PMC1145060

[46] Clark MA. Standard Protocols for the Construction of Fab Libraries. Walker JM, editor. Totowa, New Jersey: Humana Press Inc; 2002. 10.1385/1-59259-240-6:039 11968509

[47] Mouratou B, Schaeffer F, Guilvout I, Tello-Manigne D, Pugsley AP, Alzari PM, et al Remodeling a DNA-binding protein as a specific in vivo inhibitor of bacterial secretin PulD. Proc Natl Acad Sci U S A. 2007;104(46):17983–8. Epub 2007/11/07. 10.1073/pnas.0702963104 17984049 PMC2084283

[48] Galan A, Comor L, Horvatic A, Kules J, Guillemin N, Mrljak V, et al Library-based display technologies: where do we stand? Mol Biosyst. 2016. Epub 2016/06/17. 10.1039/c6mb00219f 27306919

[49] Qasba PK, Kumar S. Molecular divergence of lysozymes and alpha-lactalbumin. Crit Rev Biochem Mol Biol. 1997;32(4):255–306. Epub 1997/01/01. 10.3109/10409239709082574 9307874

[50] Worn A, Pluckthun A. Different equilibrium stability behavior of ScFv fragments: identification, classification, and improvement by protein engineering. Biochemistry. 1999;38(27):8739–50. Epub 1999/07/07. 10.1021/bi9902079 10393549

[51] Beatty JD, Beatty BG, Vlahos WG. Measurement of monoclonal antibody affinity by non-competitive enzyme immunoassay. J Immunol Methods. 1987;100(1–2):173–9. Epub 1987/06/26. 2439600 10.1016/0022-1759(87)90187-6

[52] Parizek P, Kummer L, Rube P, Prinz A, Herberg FW, Pluckthun A. Designed ankyrin repeat proteins (DARPins) as novel isoform-specific intracellular inhibitors of c-Jun N-terminal kinases. ACS Chem Biol. 2012;7(8):1356–66. Epub 2012/05/10. 10.1021/cb3001167 22568706

[53] Pluckthun A. Designed Ankyrin Repeat Proteins (DARPins): Binding Proteins for Research, Diagnostics, and Therapy. Annu Rev Pharmacol. 2015;55:489–511. 10.1146/annurev-pharmtox-010611-134654 25562645

[54] Correa A, Pacheco S, Mechaly AE, Obal G, Behar G, Mouratou B, et al Potent and Specific Inhibition of Glycosidases by Small Artificial Binding Proteins (Affitins). PLoS One. 2014;9(5). 10.1371/journal.pone.0097438 PMC4019568 24823716

