# Supplementary figures and images for: Derivative of Extremophilic 50S Ribosomal Protein L35Ae as an Alternative Protein Scaffold
Source: PLoS One. 2017 Jan 19;12(1):e0170349. doi: 10.1371/journal.pone.0170349 (PMC5245882; doi:10.1371/journal.pone.0170349)

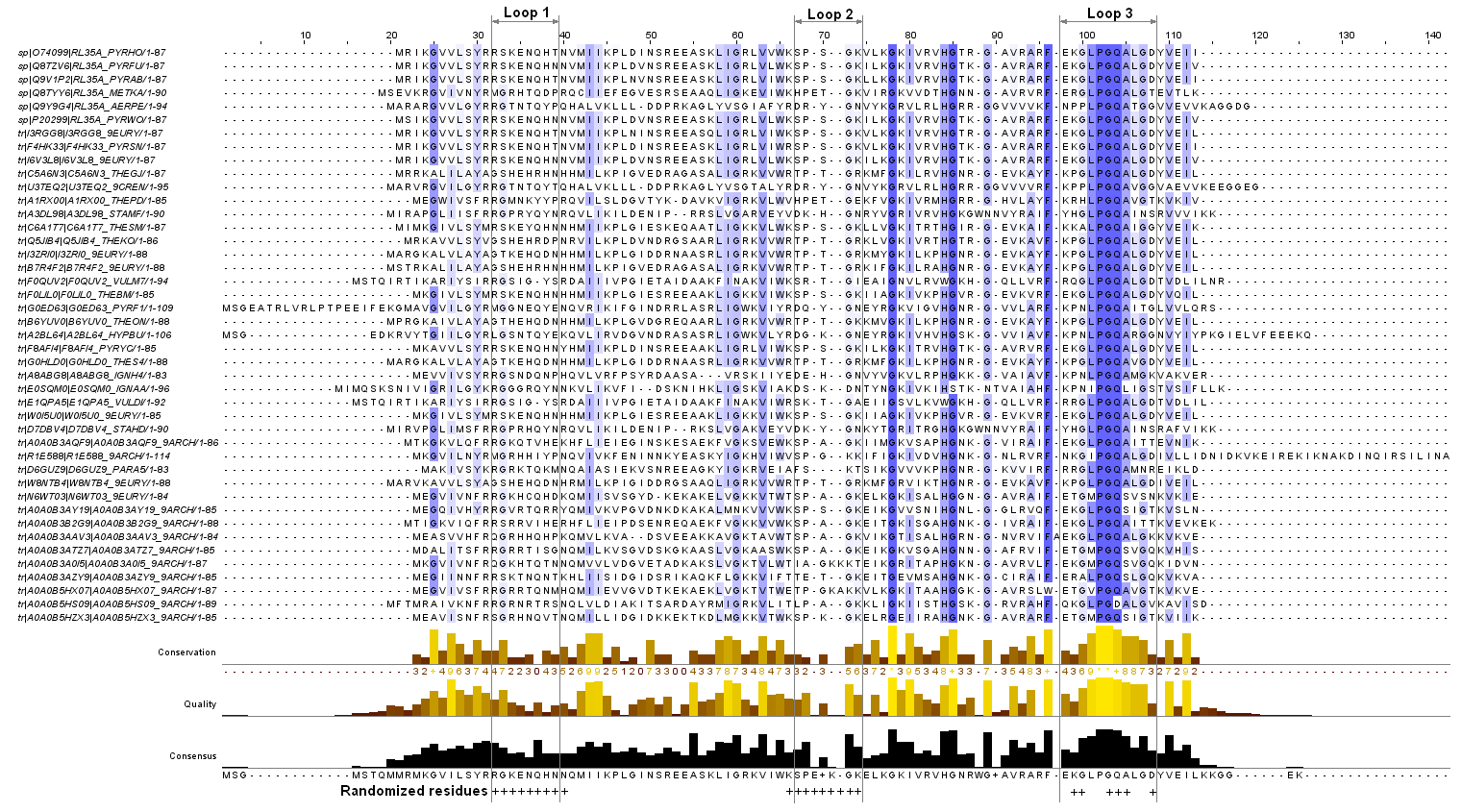

Supplement: S1 Fig — The multiple sequence alignment of the L35Ae proteins was performed using Clustal Omega v.1.2.1 algorithm [42]. Loops 1 to 3 of L35Ae from P. furiosus are indicated (refer to PDB entry 2lp6 [34], Fig 2A and 2C), as well as the residues randomized in the phage display library of L35Ae 10X (designated as ‘+’, see Fig 1). (TIF) [file pone.0170349.s001.tif]

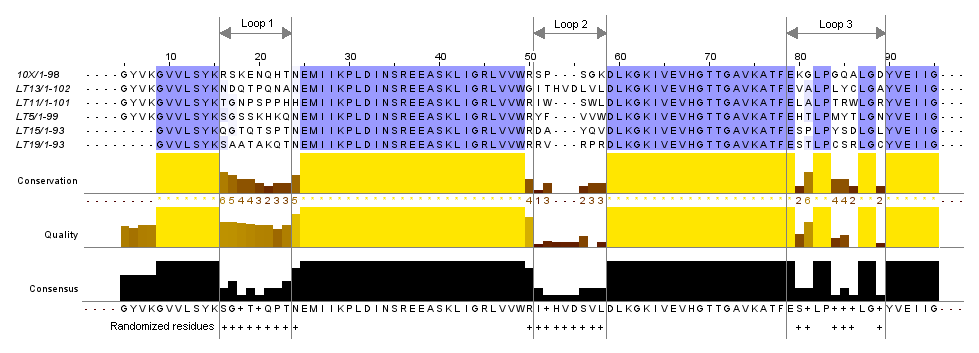

Supplement: S2 Fig — The multiple sequence alignment of the clones and the analysis of residue conservation were performed using Clustal Omega v.1.2.1 [42] and AMAS algorithms [43], respectively. Loops 1 to 3 of L35Ae from P. furiosus are indicated (refer to PDB entry 2lp6 [34], Fig 2A and 2C), as well as the residues randomized in the phage display library of L35Ae 10X (designated as ‘+’, see Fig 1). (TIF) [file pone.0170349.s002.tif]
